# The Formation of 14H-LPSO in Mg–9Gd–2Y–2Zn–0.5Zr Alloy during Heat Treatment

**DOI:** 10.3390/ma14195758

**Published:** 2021-10-02

**Authors:** Yunfang Liu, Yaqin Yang, Ming Yi, Jianmin Yu, Baocheng Li, Zhimin Zhang

**Affiliations:** College of Materials Science and Engineering, North University of China, Taiyuan 030051, China; liuyunfang0506@163.com (Y.L.); yiming@163.com (M.Y.); minyu889@163.com (J.Y.); libaocheng@nuc.edu.cn (B.L.); zhangzhimin@nuc.edu.cn (Z.Z.)

**Keywords:** Mg–9Gd–2Y–2Zn–0.5Zr alloy, solid solution treatment, second phase, 14h-lpso phase, lattice constant analysis

## Abstract

There is a new long-period stacking ordered structure in Mg–RE–Zn magnesium alloys, namely the LPSO phase, which can effectively improve the yield strength, elongation, and corrosion resistance of Mg alloys. According to different types of Mg–RE–Zn alloy systems, two transformation modes are involved in the heat treatment transformation process. The first is the alloy without LPSO phase in the as-cast alloy, and the Mg_x_RE phase changes to 14H-LPSO phase. The second is the alloy containing LPSO phase in the as-cast state, and the 14H-LPSO phase is obtained by the transformations of 6H, 18R, and 24R. The effects of different solution parameters on the second phase of Mg–9Gd–2Y–2Zn–0.5Zr alloy were studied by scanning electron microscopy (SEM), transmission electron microscopy (TEM), and X-ray diffraction (XRD). The precipitation mechanism of 14H-LPSO phase during solution treatment was further clarified. At a solution time of 13 h, the grain size increased rapidly initially and then decreased slightly with increasing solution temperature. The analysis of the volume fraction of the second phase and lattice constant showed that Gd and Y elements in the alloy precipitated from the matrix and formed 14H-LPSO phase after solution treatment at 490 °C for 13 h. At this time, the hardness of the alloy reached the maximum of 74.6 HV. After solution treatment at 500 °C for 13 h, the solid solution degree of the alloy increases, and the grain size and hardness of the alloy remain basically unchanged.

## 1. Introduction

Magnesium alloy is the lightest metal structure material in engineering application. It has broad application prospects in aerospace, automobile, and other fields due to its high specific strength, high specific stiffness, and good machinability [1,2,3,4]. However, the low absolute strength and plasticity of magnesium alloy has seriously restricted its wide application in industry [5,6,7]. Therefore, research on the improvement of the mechanical properties of magnesium alloy needs to be strengthened.

The addition of rare earth elements to a magnesium matrix can play the role of fine grain strengthening, solid solution strengthening, and precipitation strengthening, which can significantly increase the room temperature and high temperature mechanical properties of rare earth magnesium alloy [8,9]. Mg-RE alloys are a series of high-strength magnesium alloys used in engineering. When Zn is added to Mg-RE alloys, the long period stacking ordered (LPSO) phase that can effectively improve the yield strength, elongation, and corrosion resistance of magnesium alloys will appear in the alloys [10,11,12]. Wang et al. [13] studied the effect of Zn content on the microstructure and mechanical properties of hot extruded Mg-10.5Gd-5Y-0.5Zr alloy. With no Zn addition, only the β phase formed. With increasing Zn content, LPSO phase formed, and the grain was refined. Then, the strength of the alloy was improved.

The LPSO phase is a long period stacking and chemical ordering structure [14,15]. The LPSO structure in Mg-RE alloys can be divided into 10H, 14H, 18R, 24R, and others, in which H represents hexagonal stacking, and R represents rhombic stacking. Among these LPSO structures, 18R and 14H are relatively more studied [16,17]. The formation of LPSO phase structure is caused by the precipitation of supersaturated solid solution, and stacking faults are generated through the close packed hexagonal structure of Mg [18,19,20]. The LPSO phase in Mg-RE alloy has abundant structural morphologies and excellent properties. It has rapidly become the most promising structural material in the field of metal materials [21,22,23], thereby attracting great attention and leading to the upsurge of research on the atomic arrangement, formation mechanism, and mechanical properties of LPSO phase.

The conventional heat treatment processes of magnesium alloys are divided into annealing and solution and aging treatment [24,25,26]. Heat treatment can improve the mechanical properties of the alloy by controlling the microstructure of the material, such as grain size, point defects, dislocation entanglement and precipitated phase, etc. [27,28]. During the solution treatment, the coarse compound phase is partially dissolved into the matrix, the grain is refined, the stress concentration is reduced, and the strength of alloys is improved [29,30]. Hao et al. [31] carried out different heat treatment processes (water cooling and furnace cooling) on Mg_94_Zn_2.5_Y_2.5_Mn_1_(at%) alloy to obtain samples without and with 14H-LPSO phase before extrusion. Compared with the alloy without 14H-LPSO phase, the extruded alloy with 14H-LPSO phase has finer grains, lower volume fraction of dynamic recrystallization, and stronger basic texture, so it shows higher strength. Wu et al. [32] performed three solid solution treatment processes on Mg-10Gd-1Zn-0.5Zr alloy to obtain various morphologies of LPSO phase. The results show that the effect of solution treatment on the mechanical properties of block LPSO is not significant, and the formation of lamellar LPSO phase leads to a decrease in plasticity. However, in the subsequent aging process, the existence of LPSO phase can restrain the coarsening of β′ phase and improve the mechanical properties of the alloy. 

High strength Mg–Gd–Y–Zn–Zr series rare-earth magnesium alloys will generate LPSO phase after heat treatment, and the existence of LPSO phase has obvious strengthening and toughening effects on the alloy [33]. Most studies focused on the structure of LPSO phase and its effects on the microstructure and mechanical properties of the alloy [34,35]. Few studies have focused on the formation of 14H-LPSO phase in Mg–Gd–Y–Zn–Zr magnesium alloys during heat treatment. In this paper, Mg–9Gd–2Y–2Zn–0.5Zr alloy was subjected to different solid solution treatments. The solid solution strengthening effect of Gd and Y elements in Mg matrix was analysed using the volume fraction of the second phase and lattice constant, and the precipitation mechanism of 14H-LPSO phase in the process of solid solution treatment was revealed, thereby providing reference for the further application of rare earth magnesium alloy.

## 2. Materials and Experimental Methods

**Figure 1:**
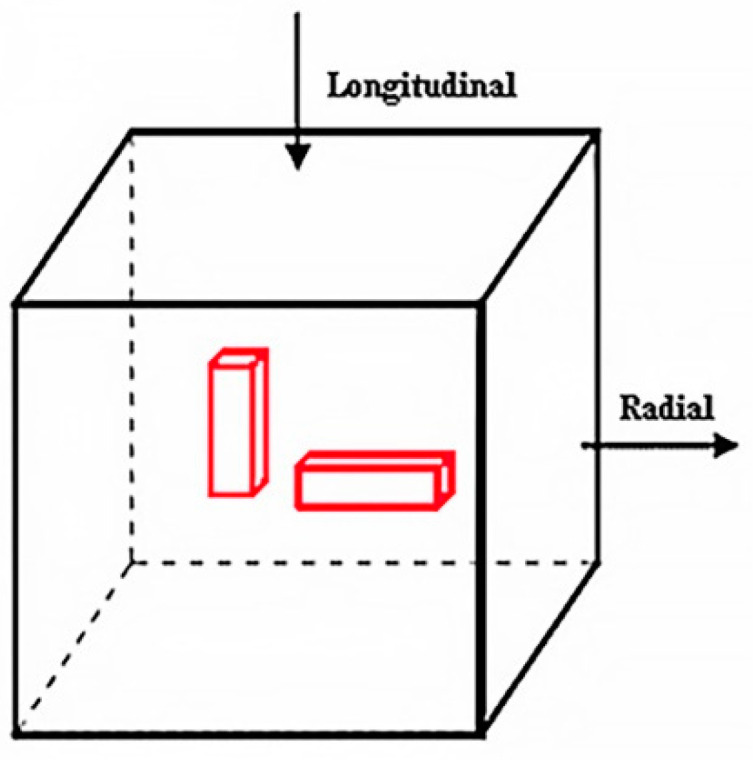
Schematic diagram of sample sampling.

The free forged Mg–9Gd–2Y–2Zn–0.5Zr alloy ingot was selected as the experimental material, and its specific chemical composition is shown in Table 1. The 200 mm cube magnesium alloy was forged on the NCHP 1250T hydraulic press, and the deformation rate was 2 mm/s. The alloy was sampled along the longitudinal direction, as shown in Figure 1.

The transformation temperature of the Mg–9Gd–2Y–2Zn–0.5Zr alloy is 528 °C. To prevent overheating, the solution temperature was 460 °C, 470 °C, 480 °C, 490 °C, and 500 °C, and the solution time was 13 h (SXW-6-6 resistance furnace was selected for solution treatment). The magnesium alloy sample was placed into the furnace at the suitable temperature. After solution treatment, the sample was cooled in hot water at 70 °C.

After grinding, polishing, and corrosion (2.5 mL anhydrous ethanol + 1 g picric acid + 18 mL distilled water + 2.5 mL glacial acetic acid), the microstructure of solution-treated magnesium alloy was observed by Zeiss Image A2m Optical microstructure (OM), Hitachi SU-5000 Scanning electron microscopy (SEM), and EDAX Inc energy dispersive spectrometer (EDS). The second phase composition was analysed by energy diffraction. The second phase was observed by JEM-2100F Transmission electron microstructure (TEM) and the selected area electron diffraction (SAED) was analysed. The magnesium alloy was analysed by Rigaku X-ray diffraction (XRD), and the Jade6.0 software was used to analyse the phase. The voltage and current were 40 KV and 40 mA, respectively, with a step size of 0.02, and the diffraction angle was in the range of 20°–80°. The volume fraction of the second phase was statistically calculated by Image Pro Plus software. UHL VMH-002VD was used to test the hardness of the alloy, and Origin2017 software was used to draw its hardness curve.

## 3. Results

### 3.1. Microstructure Analysis

**Figure 2:**
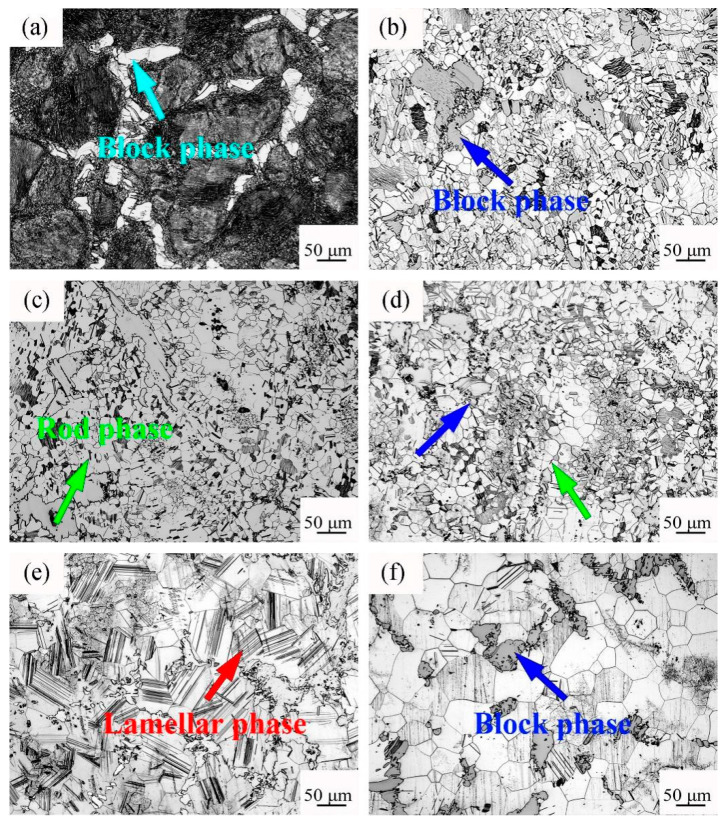
OM image of Mg–9Gd–2Y–2Zn–0.5Zr alloy after different heat treatments. (**a**) Original state, (**b**) 460 °C × 13 h, (**c**) 470 °C × 13 h, (**d**) 480 °C × 13 h, (**e**) 490 °C × 13 h, and (**f**) 500 °C × 13 h.

Figure 2 shows the microstructure of the initial state and different solution temperature. With increasing solution temperature, the grains grew obviously, and the shape of the second phase changed. In the original state, many white block phases were in the alloy and were distributed along the grain boundaries. From 460 °C × 13 h to 480 °C × 13 h, many block, rod, and dot phases were in the alloy after solution treatment, and the degree of solution was not sufficient. When the solution temperature was at 490 °C, the rod-like phase in the alloy disappeared, and many lamellar phases penetrating the grains were formed. At the solution temperature of 500 °C, the number of lamellar phases was greatly reduced, and the solution degree of the alloy was better.

**Figure 3:**
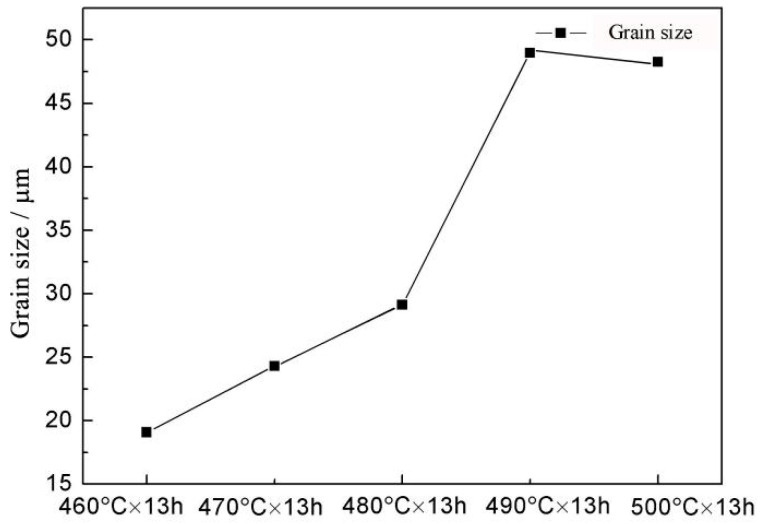
Grain size diagram of magnesium alloy at different solution temperature.

Figure 3 shows the grain size diagram of magnesium alloy at different solution temperatures. The grain size after 460 °C × 13 h solution treatment was 24.30 μm. With increasing solution temperature, the grain size of the alloy increased. When the solution temperature increased to 490 °C, the maximum grain size was 48.96 μm. After 500 °C × 13 h solution treatment, the grain size remained essentially unchanged.

### 3.2. Hardness

**Figure 4:**
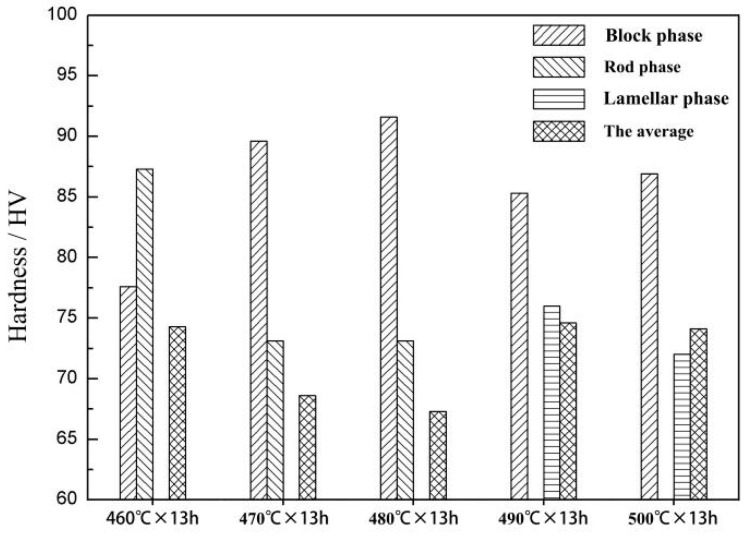
The hardness of block phase, rod phase, lamellar phase, and the average hardness of the alloy after different solution treatments.

Figure 4 shows the hardness of block rod-like and lamellar phases and the average hardness of the alloy after different solution treatments. After 460 °C × 13 h solution treatment, the rod phase had a larger hardness value than the block phase. At this time, the average hardness of the alloy was 74.3 HV. When the solution temperature increased to 480 °C, the hardness of the block phase increased, whereas the hardness of the rod phase decreased, and the average hardness of the alloy decreased to 67.3 HV. After 490 °C × 13 h solution treatment, the solid solution degree of the alloy decreased, and a large number of lamellar phases were formed. The alloy reached a peak hardness of 74.6 HV. The formation of lamellar phase was the main reason for the increase in alloy hardness. After 500 °C × 13 h solution treatment, the solid solution degree of the alloy increased, and the average hardness basically remained unchanged. The alloy in this state had good comprehensive mechanical properties. Therefore, 500 °C × 13 h was selected as the best solid solution process.

In the initial stage of solution treatment, two main factors led to a reduction in the hardness of the alloy. On the one hand, work hardening was eliminated. After solution treatment, the dislocation density decreased, and the work hardening effect was eliminated. On the other hand, the grains grew. According to the fine grain strengthening theory, the larger the grain is, the worse the hardness is. Hardness increased, because the degree of solid solution increased at high solution temperature.

## 4. Discussion

### 4.1. Different Forms of Second Phase

**Figure 5:**
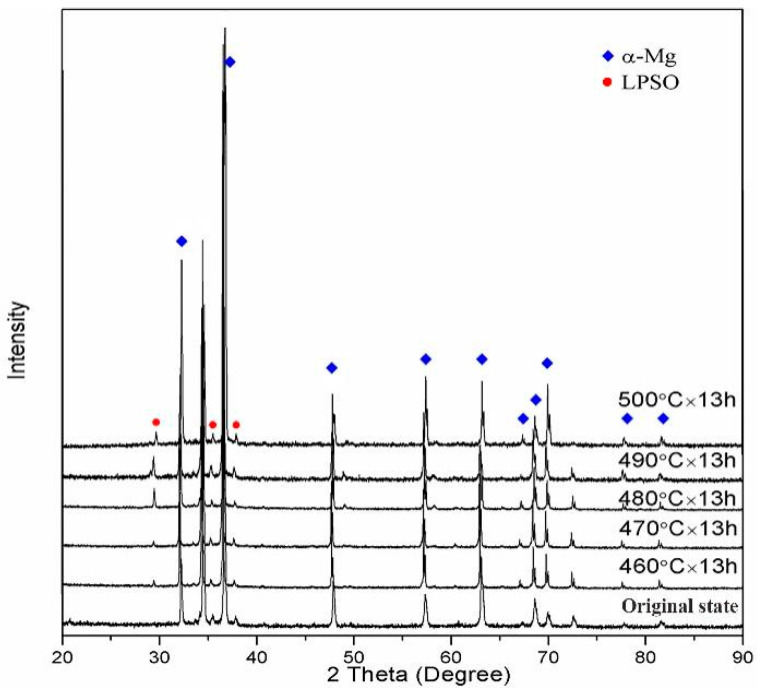
XRD of Mg alloy with a solution time of 13 h and different solution temperatures.

Figure 5 is the XRD of the Mg–9Gd–2Y–2Zn–0.5Zr alloy at a solution time of 13 h and at different solution temperatures. The internal phase composition of the alloy after solution treatment at 460 °C × 13 h, 470 °C × 13 h, 480 °C × 13 h, 490 °C × 13 h, and 500 °C × 13 h included α-Mg and LPSO phase.

**Figure 6:**
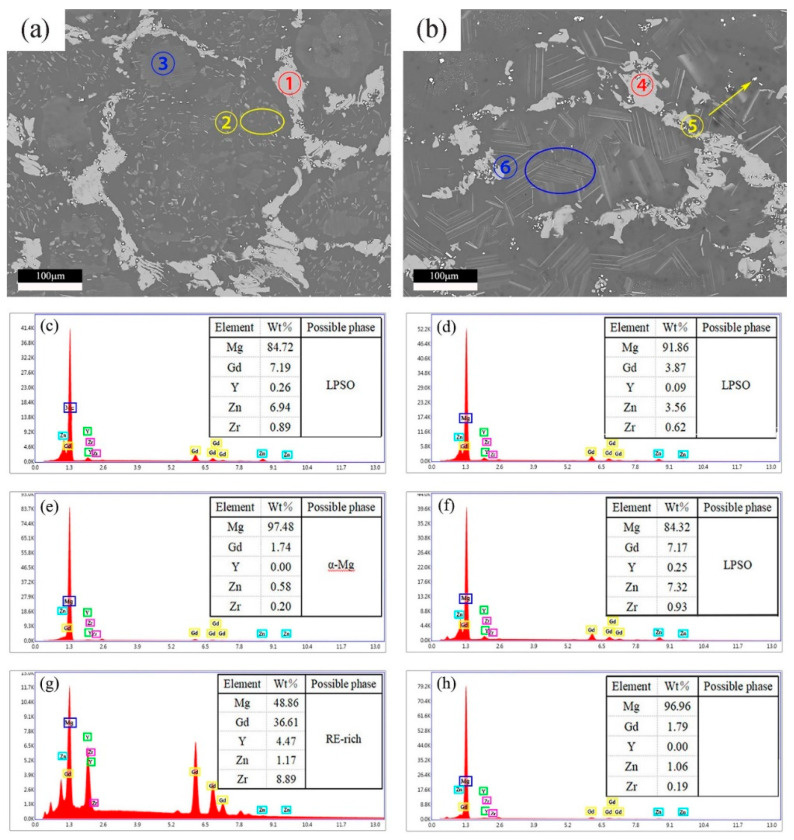
(**a**) SEM of 460 °C × 13 h, (**b**) SEM of 490 °C × 13 h (**c**) EDS results at ①, (**d**) EDS results at ②, (**e**) EDS results at ③, (**f**) EDS results at ④, (**g**) EDS results at ⑤, (**h**) EDS results at ⑥.

To analyse the composition of the second phase in different forms, EDS was used to analyse the chemical composition of different areas of the Mg–9Gd–2Y–2Zn–0.5Zr alloy after solution treatment at 460 °C × 13 h and 490 °C × 13 h. The area was selected, and its EDS result is shown in Figure 6.

The ratio of Mg:(Gd + Y): Zn at position ① was approximately 12:1:1 in Figure 6. As shown in position ②, matrixes existed in the selected region, resulting in high Mg content, due to the small size of the rod phase, and the ratio of (Gd + Y): Zn is approximately 1:1. Thus, both grey block and grey rod phases were LPSO phases. This finding was consistent with that found in Mg-Gd–Y–Zn–Zr alloy after repetitive upsetting extrusion by Zhang et al. [36]. The Gd + Y content at position ⑤ accounted for nearly 50%. Thus, it was inferred that the white dot phase was a rare earth rich phase. The lamellar phase size was too small to be analysed by EDS.

**Figure 7:**
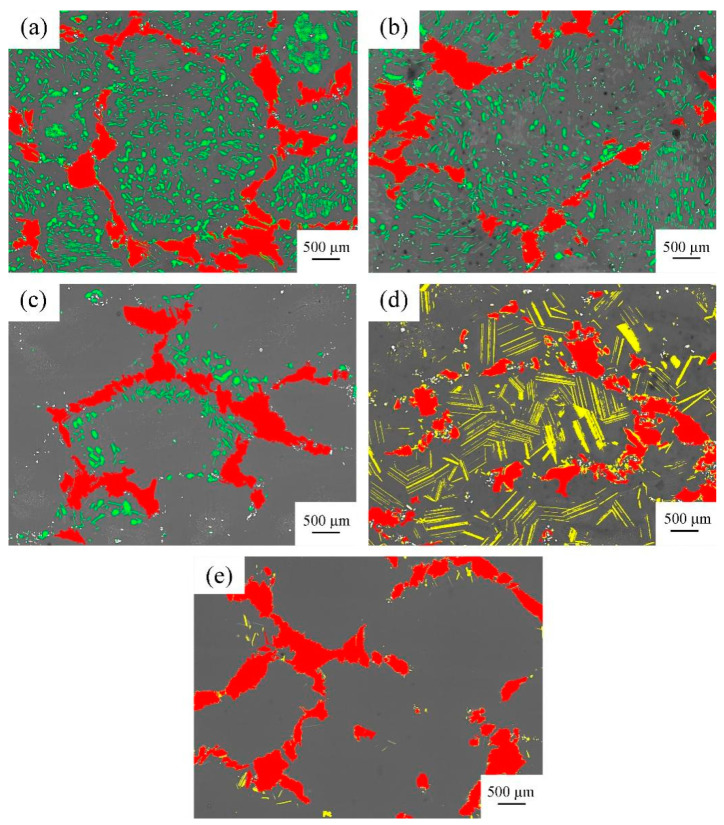
Statistical diagram of block phase, rare earth rich phase, rod phase, and lamellar phase volume fraction after different solution treatment by Image-Pro-Plus software: red is a block phase, green is a rod phase, yellow is a lamellar phase, and white is a rare earth-rich phase. (**a**) 460 °C × 13 h, (**b**) 470 °C × 13 h, (**c**) 480 °C × 13 h, (**d**) 490 °C × 13 h, and (**e**) 500 °C × 13 h.

**Figure 8:**
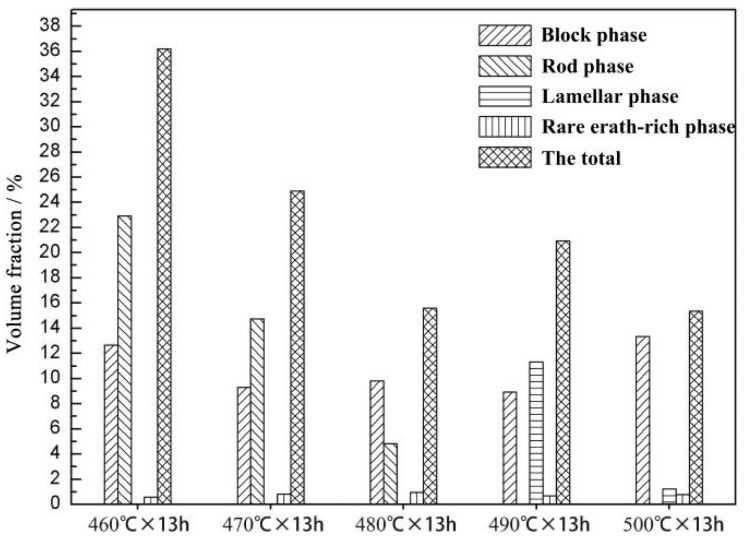
Statistical histogram of volume fraction of LPSO phase, rare earth-rich phase and total second phase.

Image-Pro-Plus software was used to analyse the block phase, rare earth-rich phase, rod phase, and lamellar phase in the SEM images of the Mg-9Gd-2Y-2Zn-0.5Z alloy, and its volume fraction was counted. The image processed by Image-Pro-Plus software is shown in Figure 7. The volume fraction statistics histogram is shown in Figure 8. The volume fraction results are shown in Table 2.

After solution treatment at 470 °C × 13 h, the volume fraction of block phase and rod phase decreased, the volume fraction of rare earth-rich phase increased, and the overall volume fraction of the second phase decreased. After solution treatment at 480 °C × 13 h, the volume fraction of the block phase and the rare earth-rich phase remained unchanged; the volume fraction of the rod phase decreased, and the overall number of the second phase decreased. After the solution treatment at 490 °C × 13 h, the volume fraction of the block phase remained unchanged, the rod phase disappeared, and many lamellar phases were formed. The total integral number of the second phase increased. After 500 °C × 13 h solution treatment, the volume fraction of the block phase increased, the volume fraction of the lamellar phase decreased, and the total integral number of the second phase decreased. The volume fraction of the second phase in the alloy was smallest after solution treatment at 500 °C × 13 h. Therefore, 500 °C × 13 h was the best solution treatment process.

By comparing 480 °C × 13 h and 490 °C × 13 h solution treatments, we determined that, as the solution temperature increased, the volume fraction of the block phase decreased from 9.83 to 8.93%; the rod phase decreased from 4.81% to 0; the lamellar phase increased from 0 to 11.32%; and the rare earth-rich phase increased from 0.84 to 0.97%.

The volume fraction of the block, the rod, and the rare earth-rich phases changed by 5.58%, and the volume fraction of the lamellar phase decreased from 11.32 to 1.32%, which was a total decrease of 10%. From the change of the volume fraction of phase, we can see that the lamellar phase was not transformed from the block phase, rod phase, and rare earth-rich phase. The lamellar phase was derived from the inside of a Mg matrix.

### 4.2. LPSO Structure

**Figure 9:**
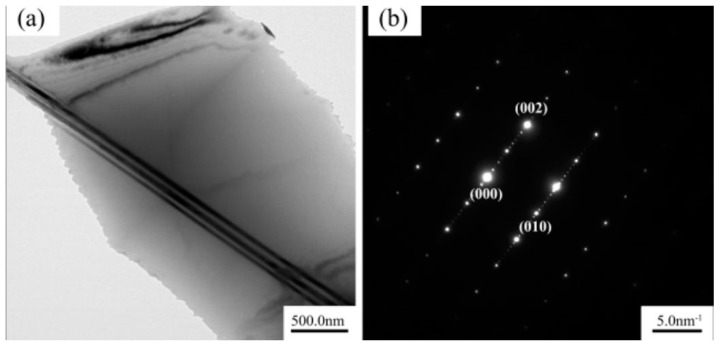
(**a**) TEM bright field image (**b**) SAED.

Figure 2 and Figure 7 show that many lamellar phases were formed after 490 °C × 13 h solution treatment. The structure of the lamellar phase that was formed after 490 °C × 13 h solution treatment was further confirmed through TEM, and the diffraction pattern analysis was carried out. The diffraction pattern of the selected area in Figure 9 showed that 14 diffraction spots were present from (000) to (002), which confirmed that the lamellar phase was the 14H-LPSO phase.

The formation of LPSO phase was due to the change in stacking fault energy caused by the addition of Zn. Gd, Y, and Zn gathered between stacking faults, and the long period stacking ordered structure (LPSO phase) was formed when certain kinetic and thermodynamic conditions were reached [37,38]. Wu et al. [39] carried out solid solution treatment on Mg_96.5_Gd_2.5_Zn_1_ alloy with a 14H-LPSO structure. At 773 k, a 14H-LPSO structure can appear in the matrix and be transformed by the β phase with fcc structure. Yoshihito Kawamura and Michiaki Yamasaki [40] studied the LPSO phase in Mg_97_Zn_1_RE_2_ (RE = Y, La, Ce, Pr, Nd, Sm, Eu, Gd, Tb, Dy, Ho, Er, Tm, Yb) alloy and found two sources of LPSO phase, namely the casting and heat treatment processes. The 14H-LPSO phase was formed in the alloy after solution treatment. The main reason for the formation of the 14H-LPSO phase was that Zn element reduced the stacking fault energy of the alloy at high temperatures, making it easy for the alloy to form stacking faults. In addition, Gd elements were concentrated in the stacking faults, resulting in the formation of the 14H-LPSO phase.

### 4.3. Formation of 14H-LPSO

The LPSO phase, which contains Mg, Gd, Y, and Zn elements, was formed in Mg–9Gd–2Y–2Zn–0.5Zr alloy after solution treatment [41,42]. The atomic radii of Gd and Y were 2.54 and 2.27 A, respectively, which were larger than that of magnesium (1.60 A). They were dissolved in a Mg matrix to increase lattice distortion. The atomic radius of Zn was 1.34 A, which was close to the atomic radius of Mg. Zr is often used to refine Mg-RE alloy grains and does not participate in and hinder the decomposition of Mg-RE alloy solid solution. Therefore, XRD is used to further study the change of the matrix lattice constant after solution treatment. It is possible to research the solution strengthening effect of the Gd and Y elements on the magnesium-based alloy by measuring the 2θ angle and the interplanar spacing of the Mg–9Gd–2Y–2Zn–0.5Zr alloy after different solution treatments [43].

The Brag Equation, 2dsinθ = nλ, shows that the value of d was inversely proportional to the angle θ; when the value of d increased, the angle θ decreased. As the solution temperature increased, Gd and Y elements dissolved into the matrix, and the atomic radii of Gd and Y were larger than that of Mg atoms. Therefore, the interplanar spacing in the a-Mg matrix increased, the value of d increased, and the 2θ angle decreased.

**Figure 10:**
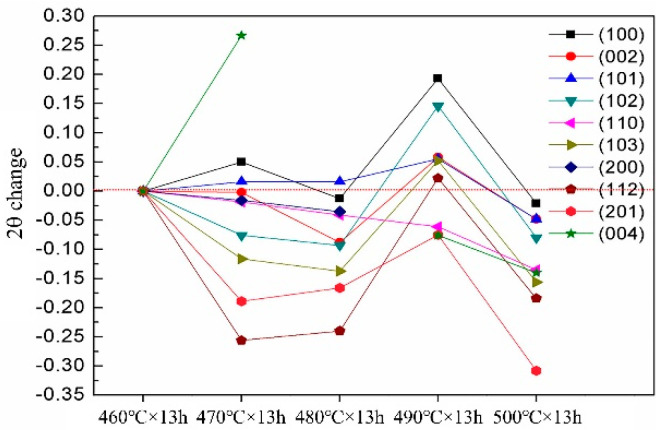
2θ change.

**Figure 11:**
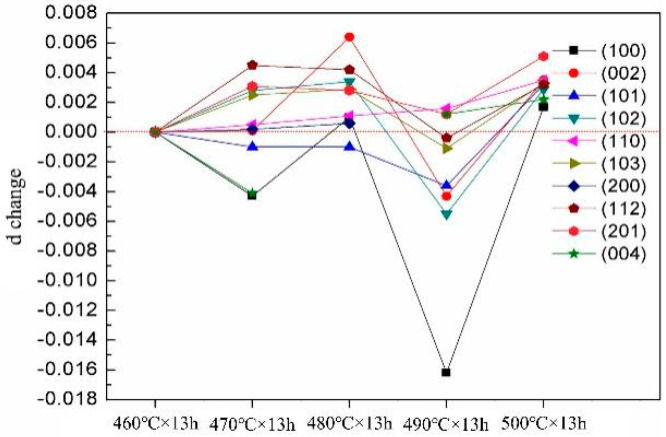
d change.

Table 3 shows the 2θ angle and interplanar spacing d values of the Mg–9Gd–2Y–2Zn–0.5Zr alloy with different solution treatments. To observe the changes of 2θ angle and interplanar spacing d value intuitively, the 2θ angle and interplanar spacing d value of Mg–9Gd–2Y–2Zn–0.5Zr alloy after solution treatment at 460 °C × 13 h were taken as the standard, and the 2θ angle and interplanar spacing d values after other solution treatments were compared with them, as shown in Figure 10 and Figure 11.

Table 3 and Figure 10 and Figure 11 show that the 2θ angle of the alloy decreased with increasing solution temperature from 460 to 480 °C. At 490 °C, the 2θ angle, which was greater than and close to the 2θ angle of 460 °C, did not decrease but increased. The 2θ continued to decrease at 500 °C and was lower than that at 460 °C. The d value of the alloy increased with increasing solid solution temperature from 460 to 480 °C. The d, which was less than and close to the d value of 460 °C value, did not increase but decreased at 490 °C. The d value continued to increase at 500 °C and was higher than that at 460 °C. At 460, 470, and 480 °C, with increasing solution temperature, the 2θ angle decreased, and the value of d increased. With increasing solid solution temperature, a large amount of second phase dissolved into the matrix, and the contents of Gd and Y in the matrix increased. At this time, the larger radius of Gd and Y atoms increased the lattice distortion of the alloy, and finally, the 2θ angle decreased, and the d increased. An abnormal phenomenon occurred at 490 °C, that is, the 2θ angle increased, which was greater than and close to the 2θ of 460 °C. The d value decreased, which was less than and close to the d of 460 °C. The Gd and Y elements all precipitated from the matrix to form the 14H-LPSO phase. At 500 °C, the 2θ angle decreased and was lower than the 2θ of 460 °C. The d value increased and was greater than the d of 460 °C. The 14H-LPSO phase dissolved into the matrix, that is, Gd and Y elements dissolved into the matrix.

In recent years, scholars have conducted in-depth research on the microscopic formation mechanism of LPSO phase. Through theoretical calculations and experimental observations, Zhu et al. [44] found that the stacking fault energy of pure magnesium was between 30 and 80 mJ/m^2^. After adding Zn and Y, the stacking fault energy of Mg-Y-Zn alloy was reduced to 0.9–1.8 mJ/m^2^. The nature of LPSO phase was an orderly stacking of stacking faults, and the low stacking fault energy of Mg-Y-Zn alloy facilitated the formation of stacking faults. Moreover, they studied Mg-Y-Zn alloys using bright-field of dual-beam and dark-field of weak-beam imaging techniques and found that a large number of stacking faults were formed in the alloy first, and Y and Zn atoms diffused to form an LPSO structure. Thus, they believed that the simple transformation of LPSO phase was a diffusion-displacement phase transition. Kishida et al. [45,46,47,48,49] used HAADF-STEN technology to study the formation mechanism of LPSO phase in a Mg-Gd-Al alloy and found that Gd and Al atoms were enriched in four consecutive hcp atomic layers. Subsequently, a stacking fault that broke the original hcp structure was introduced in the four consecutive atomic layers enriched with Gd and A1 atoms, leading to the formation of L1_2_ fcc stacking blocks. Among them, the Gd and Al atoms in the stacked blocks formed Al_6_Gd_8_ atomic groups. The Gd and Al atoms are arranged in an orderly manner between the layers with the lowest energy, so that the L1_2_ fcc stacking blocks are constantly “roughened”. Finally, the stacked blocks formed the LPSO phase with the lowest energy. The research of Zhu and Kishida showed that, in the process of forming the LPSO phase, solute atoms, such as Gd and Y, were precipitated from the Mg matrix.

## 5. Conclusions

In this paper, the effects of solution temperature on the microstructure and mechanical properties of a Mg–9Gd–2Y–2Zn–0.5Zr alloy were studied when its time was 13 h. The change of the second phase with increasing solution temperature was analysed in detail, and the precipitation mechanism of lamellar LPSO phase during solution treatment was revealed. The specific conclusions were as follows:

(1) When the solution temperature was 490 °C, a large number of lamellar LPSO phases were formed in the alloy, and the grain size increased. The number of lamellar phases was greatly reduced, and the grain size was almost unchanged when the solution temperature was raised to 500 °C.
(2) After 490 °C × 13 h solution treatment, the average hardness of the alloy was the highest, which is due to the great contribution of the lamellar structure. After 500 °C × 13 h solution treatment, although the hardness decreases slightly, the grain size of the alloy decreases, the volume fraction of the second phase is the least, and the solid solution degree of the alloy is sufficient. Therefore, 500 °C × 13 h is comprehensively considered is the best solution treatment process.
(3) After 490 °C × 13 h solution treatment, the lamellar phase was 14H-LPSO phase according to the diffraction pattern analysis. Through the analysis of the volume fraction of the second phase and the lattice constant, it was proven that Gd and Y elements were derived from the Mg matrix, and then, the LPSO phase was formed.

## Figures and Tables

**Table 1:** Chemical composition (wt%) of a Mg–9Gd–2Y–2Zn–0.5Zr alloy.

Mg	Gd	Y	Zn	Zr
Bal	9.2	1.9	1.8	0.5

**Table 2:** Volume fraction statistics of block phase, rod phase, lamellar phase, rare earth-rich phase, and total second phase (%).

Sample	Block Phase	Rod Phase	Lamellar Phase	Rare Earth-Rich Phase	The Total
460 °C × 13 h	12.67	22.93	0.00	0.58	36.18
470 °C × 13 h	9.30	14.76	0.00	0.84	24.90
480 °C × 13 h	9.83	4.81	0.00	0.97	15.61
490 °C × 13 h	8.93	0.00	11.32	0.68	20.93
500 °C × 13 h	13.35	0.00	1.23	0.79	15.37

**Table 3:** The 2θ and d of Mg–9Gd–2Y–2Zn–0.5Zr alloy by different solution treatment.

(hkl)	460 °C × 13 h		470 °C × 13 h		480 °C × 13 h		490 °C × 13 h		500 °C × 13 h	
	2θ	d	2θ	d	2θ	d	2θ	d	2θ	d
(100)	32.166	2.7806	32.216	2.7763	32.253	2.7816	32.359	2.7644	32.145	2.7823
(002)	34.421	2.6034	34.419	2.6035	34.333	2.6098	34.479	2.5991	34.373	2.6069
(101)	36.577	2.4547	36.593	2.4537	36.593	2.4537	36.632	2.4511	36.529	2.4579
(102)	47.871	1.8987	47.795	1.9015	47.778	1.9021	48.017	1.8932	47.791	1.9016
(110)	57.428	1.6033	57.409	1.6038	57.387	1.6044	57.367	1.6049	57.293	1.6068
(103)	63.159	1.4709	63.043	1.4734	63.022	1.4738	63.211	1.4698	63.003	1.4742
(200)	67.359	1.3891	67.343	1.3893	67.324	1.3897				
(112)	68.780	1.3638	68.524	1.3683	68.540	1.3680	68.802	1.3634	68.596	1.3670
(201)	70.048	1.3422	69.859	1.3453	69.882	1.3450	69.973	1.3434	69.740	1.3473
(004)	72.468	1.3032	72.735	1.2991			72.392	1.3044	72.328	1.3054

## Data Availability

Data are contained within the article.
